# The resting calcaneal stance position (RCSP): an old dog, with new tricks

**DOI:** 10.1007/s00431-023-05354-w

**Published:** 2023-12-16

**Authors:** Carlos Martinez-Sebastian, Gabriel Gijon-Nogueron, Laura Ramos-Petersen, Cristina Molina-Garcia, Rubén Sánchez-Gómez, Angela M. Evans

**Affiliations:** 1 Department of Nursing and Podiatry, Faculty of Health Sciences, University of Malaga, 29071 Malaga, Andalucia, Spain; 2 IBIMA, University of Málaga, Malaga, Spain; 3 Department of Podiatry, Universidad Católica San Antonio de Murcia, Campus de los Jeronimos, 30107 GuadalupeMurcia, Spain; 4 Nursing Department, Faculty of Nursing, Physiotherapy and Podiatry, Universidad Complutense de Madrid, 28040 Madrid, Spain; 5 Discipline of Podiatry, College of Science, Health, and Engineering, La Trobe University, Melbourne, VIC Australia

**Keywords:** Flat foot, Children, Resting calcaneal stance position, Foot posture index

## Abstract

The objective of this study was to establish the accuracy of the resting calcaneal stance position (RCSP) for the assessment of flat foot (FF) in children, aligned to the validity of the foot posture index (FPI). The RCSP cut-off point was explored, in context of both FF prevalence and the relationship between FF and body weight. A total of 205 healthy children, aged 5 to 10 years, participated in a cross-sectional study. Correlation was performed between RCSP and FPI. ROC curve technique was calculated to assess differentiation between groups. A score equal to or greater than 7 on the FPI was used as the ‘gold standard’ for analysis. The correlation between FPI and RCSP was significant (*r* = 0.63; *p* < 0.01). The discrimination score on the ROC curve (6 points/degrees) shows that the model can be used to identify FF through RCSP, with a sensitivity of 67% and specificity of 85% returned.

*  Conclusion*: The results of this study indicate the role of RCSP for simple, accessible and quick screening of paediatric FF. This is especially pertinent for non-podiatric healthcare professional without specialised paediatric foot knowledge.

**Table:** 

**What is Known:** *• Most children develop a normal arch quickly, and flat feet usually resolve on their own between 2 and 6 years of age.* *• The measurement used to diagnose flat foot in children must be accurate, consistent, and valid to characterize the standard foot position. The Resting Calcaneal Stance Position (RCSP) is another widely used measure to evaluate the position of the flat foot in children.*	
**What is New:** *• The RCSP cut-off point 6 shows a sensitivity of 67% and a specificity of 85% thanks to the FPI as the Gold standard.* *• The RCSP is useful for health professionals who are not specialised in pediatric foot health. The RCSP is useful to detect flat foot in children.*

**What is Known:**

*• Most children develop a normal arch quickly, and flat feet usually resolve on their own between 2 and 6 years of age.*

*• The measurement used to diagnose flat foot in children must be accurate, consistent, and valid to characterize the standard foot position. The Resting Calcaneal Stance Position (RCSP) is another widely used measure to evaluate the position of the flat foot in children.*

**What is New:**

*• The RCSP cut-off point 6 shows a sensitivity of 67% and a specificity of 85% thanks to the FPI as the Gold standard.*

*• The RCSP is useful for health professionals who are not specialised in pediatric foot health. The RCSP is useful to detect flat foot in children.*

## Introduction

The issue of flat feet (FF) in children is a long-debated issue, with uncertainty and confusion regarding feet considered normal or abnormal, when to treat or simply observe, how best to treat without surgery and when and how to intervene surgically [1–3]. When analysing a child with FF, the agreed primary differentiation is between the physiological and pathological forms. Despite this, there is no universally accepted definition for FF in children, although consistent features include a valgus heel and a flattened medial longitudinal foot arch [4].

In essence, there are two types of FF: flexible and rigid. A flexible flat foot is expected in children, yet a common concern for parents. It is characterized by the reduction of the medial arch with weight-bearing [5]. In most cases, flat feet between 2 and 6 years of age evolve into a normal arch [6]. In infants, many foot bones are ossifying, ligaments are very flexible, fatty tissue resides through the non-weight loaded foot arch, and muscle control is still in the process of neuro-maturation [7, 8]. Rigid FF are uncommon, affecting less than 1% of the population [9]. Rigid FF are characterised by having an unchanging low/flat medial foot arch in both weight-bearing and non-weight-bearing states [5].

Paediatric FF is usually physiological and painless [10]. Pathological FF in children do occur and can be variably symptomatic, with the potential for long-term problems, such as posterior tibial tendon dysfunction and lower limb pain [11]. Such cases may also be associated with foot anomaly, such as bunions and hammertoes [12].

Hence, whilst most paediatric FF indicate normal physiology, it is important to identify the minority of pathological cases, so that intervention can be implemented to avert further pain and functional limitations [2]. Generally, conservative measures precede surgical approaches, with foot orthoses and footwear being mainstays. In the uncommon FF that are not managed conservatively, surgical interventions are considered, as is often the case for rigid FF [4, 13].

The assessment of children’s FF may involve several methods, such as X-rays, static measures of foot position and footprint analysis [14]. As with all useful measures, those used to assess FF must be consistent and valid for characterization from a standard foot position.

The diversity of testing approaches has resulted in wide variation in the reported incidence of paediatric FF, ranging from 0.6 to 77.9% [15]. A systematic review addressing paediatric FF measures [14] reported that most information influencing clinicians to both evaluate and treat FF are based on unsubstantiated measures. In contrast, the foot posture Index (FPI) is demonstrably reliable and valid as an established tool to evaluate foot position [16]. The FPI was developed over 20 years ago; this tool identifies the morphology in the 3 cardinal axes of the foot as its definition justifies the FF, differentiating itself from tests such as footprint indices [17]. Use of the FPI allows doctors to track change from baseline as children grow or as a presentation progresses. The FPI is a comprehensive 6-item measure, which evaluates the three-dimensionality of the foot through palpation and observation. The FPI enables easy and non-invasive classification to five foot posture categories, ranging from highly supinated to highly pronated (known as FF) [17]. Gijon-Nogueron et al. [16] compiled large-scale paediatric normative data, finding that a FPI total score ≥  + 7 is considered FF [18].

The resting calcaneal stance position angle (RCSP) is a single, one-plane, clinical measure, which continues in wide use, to assess the valgus heel facet of FF [19]. Previous research [20] has correlated FPI with RCSP to simplify the clinical detection of paediatric FF using a single measure.

There is increased awareness from a range of medical professionals and the community, regarding wider health and the importance of walking [2], and paediatric foot concerns present to many health and medical professionals. As a concept, RCSP is an accessible FF screening measure that is simple and quick to apply in the clinical setting, when the doctor or paediatrician has little time and non-particular foot knowledge.

The aims of this study were to establish whether the RCSP may align with the validated FPI in the clinical detection of paediatric FF; relate RCSP cut-off points with body weight; and avail a simple, non-invasive, low-cost, clinical measure for use by a range of health and medical professionals to screen for paediatric FF, when not specialised in paediatric feet nor gait.

## Materials and methods

### Ethical approval

This study was carried out in full compliance with the ethical principles established in the Declaration of Helsinki regarding medical research with human participants. In addition, it received approval from the Ethics Committee, specifically from the Ethics Committee of the Universidad Católica San Antonio de Murcia under the reference CE112104. Ethical approval was obtained from children’s parents who signed an informed consent.

### Participants

In this cross-sectional study, a total of 205 children, aged from 5 to 10 years, were involved as research participants. The measurements occurred during 2022, specifically from January to June at the San Francisco de Asís School in Lorca, Murcia (Spain).

Inclusion criteria were children aged 5 to 10 years, not experiencing foot pain during the evaluation and informed permission from parents or guardians. Exclusion criteria were a rigid FF, congenital structural anomalies of the foot or ankle, cerebral palsy, surgery to the foot or lower extremity and genetic, neurological or muscular diseases.

### Procedure

Demographic data was collected, including: sex, age, weight and height. The children wore comfortable sports clothing, specifically shorts, and were barefoot during the measurements. The children were asked to relax, and all measurements of joint mobility were carried out without causing any discomfort or pain.

Body mass index (BMI) was calculated from participants’ height and weight (BMI = weight (kg)/height(m)^2^). Given Spanish participants, BMI classification per Orbegozo [21] was used to group participants according to BMI and age. This categorization establishes four different ranges as follows: low weight, BMI is below the 3rd percentile (P < 3); normal weight, BMI is between 3rd and 90th percentiles (P: 3–90); overweight, BMI is between the 90th and 97th percentiles (P: 90–97); and obesity, percentile greater than 97th (P > 97).

Table 1 shows the children classified by BMI group, and by age.

**Table 1:** Descriptive statistics (*n* = 196)

			FPI (points)			RCSP (degrees)		
Age	*n*		Mean (*SD*)		*p*-value	Mean (*SD*)		*p*-value
	Male	Female	Male	Female		Male	Female	
5	14	17	4.5 (2.5)	3.2 (3.2)	.220	4.4 (2.4)	4.3 (2.6)	.11
6	25	23	4(3)	3.9 (2.8)	.922	5.8 (1.8)	3.8 ¿2.1)	.758
7	16	18	5.3 (2.6)	4.1 (3.2)	.268	4.4 (2.7)	4.1 (3.1)	.514
8	8	25	3 (2.6)	2.8(2.5)	.879	4.6 (2.3)	4 (3)	.126
9	12	33	3.4(3.5)	4.4 (2.4)	.273	4 (2.1)	5 (2.5)	.279
10	3	2	4 (3,6)	6(1.4)	.361	3.6 (2.3)	4 (0)	.668
Total	78	118	4.1 (2.9)	3.8 (2.8)	.205	4.4 (2.4)	4.3 (2.6)	.181

*FPI* foot posture index, *RCSP* rest calncaneal stance position

Two experienced researchers performed all measurements on both feet, of each child.

Foot position was measured with the FPI, with participants barefoot, standing relaxed, and examiners using visual observation and manual manipulation. The six criteria were evaluated (talar head palpation, supramalleolar and inframalleolar curvature, frontal plane position of the calcaneus, talonavicular prominence, medial arch congruence and forefoot abduction/adduction). Each criterion was scaled from + 2 (pronated) to − 2 (supinated), with 0 representing a mid-point position. Criterion scores were summed to a total score, which classified foot posture categories, ranging from highly supinated to highly pronated [17].

Inter-observer reliability of the FPI, in children aged 5 to 16 years has been shown to be very high, with a consistent weighted Kappa coefficient of 0.86 [22], in addition to excellent intra-observer reliability (*ICC* = 0.93–0.94) [23].

The RCSP was evaluated using the standard method [24]. Measurements were taken from both feet of each participant. On each process, the participant was lying face down with their ankle flexed to 90°, so that their heel was aligned perpendicularly with their leg in the sagittal plane. Next, the posterior aspect of the calcaneus of both feet was sectioned with a fine marker. The angle of the calcaneus bisection line and that perpendicular to the floor was measured in degrees. Intra-observer reliability for the RCSP in the paediatric population showed a good weighted kappa value (*Kw* = 0.61 and 0.90) [25].

### Statistical analysis

All statistical analyses were carried out using SPSS version 29 (IBM SPSS Statistics SPSS Inc., 2022]. An exploratory analysis of the data was performed, encompassing descriptive statistics for FPI, RCSP, age, gender and BMI. Normality of data distribution (Kolmogorov–Smirnov test) and data homogeneity (Levene) found that sample data were not normally distributed, directing descriptive statistics to characterise the FPI and RCSP. Accordingly, Spearman’s (rho) correlation coefficient was determined for continuous variables, and the chi-square test was used for qualitative variables. To preserve the Independence of data [26], based on the strong correlation between FPI scores for left and right feet in previous studies [16], statistical analysis addressed the left foot, chosen at random [27].

A paired samples *t*-test was used to compare the FPI and RCSP between left and right sides.

The ROC curve technique was used to determine how effective the FF is in differentiating between groups. This technique revealed the ability of FF to correctly detect positive and negative cases at various levels, in addition to evaluating overall performance using the area under the curve (AUC) [28]. The cut-off point represents the strongest discrimination between children with FF and straight feet. An FPI score ≥  + 7 was used as the ‘gold standard’ for analysis. The level of statistical significance was set at *p* < 0.05.

Reliability analysis was assessed by calculating the intraclass correlations (*ICC*s) for RCSP. *ICC* across the same-subject repeated measures trials were calculated for each of the two examiners (intra-rater) in 25 children and between the two examiners (inter-rater) in 25 children.

## Results

A total of 196 children were considered in the final analysis, as nine were excluded due to the pain during examination. The average age of participants was 7.6 years (range, 5 to 10 years); 60.2% were girls and 40.8% were boys. The average body mass index (BMI) was 17.8 kg/cm^2^, with no statistically significant differences by gender (*p* = 0.40) or age (*p* = 0.01).

Regarding laterality, there were no significant differences between left and right-sides for either the FPI or the RCSP.

Overall, FPI values were slightly higher in boys compared to girls: 4.1 (2.9) versus 3.8 (2.8) (*p* = 0.205). The RCSP was slightly higher in boys compared to girls: 4.4 (2.2) degrees versus 4.3 (2.6) degrees (*p* = 0.181). Descriptive statistics of the FPI and RCSP are presented in Table 1.

Bivariate analyses were performed to determine the relationships between the FPI and RCSP, in both feet. The correlation between FPI and RCSP was significant (*R* = 0.63; *p* < 0.01).

### Identification of the cut-off point and predictive values

**Fig. 1:**
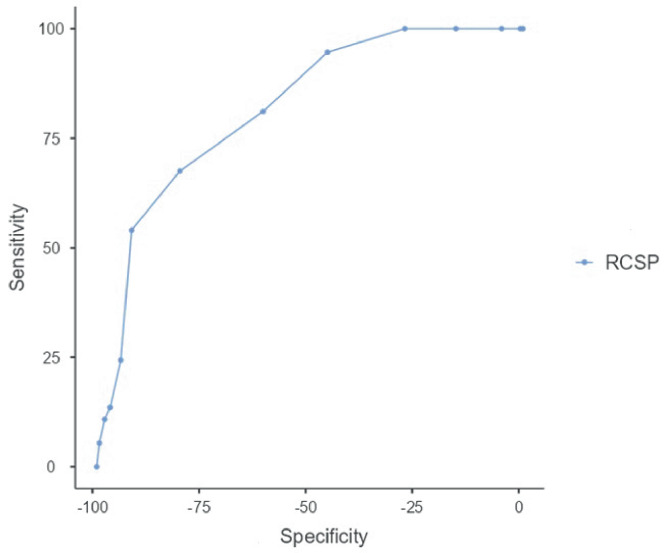
ROC curve for the RCSP. ROC curve: combined. RCSP — rest calcaneal stance position

The detection of FF using RCSP, used a determined cut-off point. All children were evaluated by expert examiners for the FPI (*AUC* 0.81, *p* < 0.001). The point on the ROC curve closest to both axes determines the appropriate score for sensitivity and specificity. The discrimination score on the ROC curve (6 points/degrees) shows that the model can be used to identify FF through RCSP (Fig. 1). Analysis by gender found no differences in the cut-off point. The maximum related Youden index was 0.48 and the cut-off point was 6, a score ≥ 6 detected FF using the RCSP (Table 2).

**Table 2:** Sensitivity and 1-specificity for RCSP

Cut-point	Sensitivity (%)	Specificity (%)	*PPV* (%)	*NPV* (%)	Youden’s index	*AUC*	Metric score
6	67.57%	80.5%	44.64%	91.43%	0.481	0.818	1.48
7	54.05%	91.82%	60.61%	89.57%	0.459	0.818	1.46

*PPV* positive predictive values, *NPV* negative predictive values, *AUC* area under the curve

Once the cut-off point for FF detection using the RCSP (as 6 degrees) was determined, a prevalence study according to both age and sex was carried out for the FPI (Table 3).

**Table 3:** Relationship between individual variables and the appearance of flat feet identified by the FPI or RCSP diagnostic methods

Variable	FPI		RCSP
Age			
5 (*n* = 31)	(25.8%)		(41.9%)
6 (*n* = 48)	(16.7%)		(25%)
7 (*n* = 34)	(23.5%)		(29.4%)
8 (*n* = 33)	(24.2%)		(19.2%)
9 (*n* = 45)	(15.6%)		(20%)
10 (*n* = 5)	(20%)		(0%)
Total (*n* = 196)	(18.9%)		(29.6%)
Sex			
Male (*n* = 116)	(20.5%)		(29.5%)
Female (*n* = 92)	(17.8%)		(28%)
BMI			
Underweight (P < 3) (*n* = 0)	(0%)		(0%)
Normal (P3–90) (*n* = 145)	(19.3%)		(18%)
Overweight (P90–97) (*n* = 19)	(15.8%)		(31.6%)
Obesity (P > 97) (*n* = 32)	(28.1%)	(34.4%)	

*BMI* body mass index, *FPI* foot posture index, *RCSP* rest stance position

Our results for BMI and waist-hip ratio (WHR), found a majority of participants with normal weight (74%), divided into straight feet (71.7%) and FF (28.3%). The percentage of overweight was 9.7%, which subdivided into straight feet (68.4%) and FF (31.6%). Obese students were 16.3%, divided into straight feet (65.6%) and FF (34.4%).

Overall, there was no significant difference in the prevalence of FF between those with different weight classifications (χ^2^ = 50.8; *p* = 0.776). However, the 8-year-old participants did return significant differences between groups (χ^2^ = 10.43; *p* = 0.005) (Table 4).

**Table 4:** Relationship between BMI and foot posture

Age			Percentile			Total
			Normal *n* (%)	Overweight *n* (%)	Obesity *n* (%)	
5.00	RCSP	Straight	14 (77.8)	3 (16.7)	1 (5.6)	18
		FF	11 (84.6)	0 (0)	2 (15.4)	13
	Total		25 (80.6)	3 (9.7)	3 (9.7)	31
6.00	RCSP	Straight	28 (77.8)	2 (5.6)	6 (16.7)	36
		FF	9 (75)	1 (8.3)	2 (16.7)	12
	Total		37 (77.1)	3 (6.3)	8 (16.7)	48
7.00	RCSP	Straight	18 (75)	1 (8.3)	5 (16.7)	24
		FF	8 (80(	1 (10)	1 (10)	10
	Total		26 (76.5)	2 (5.9)	6 (17.6)	34
8.00	RCSP	Straight	22 (81.5)	3 (11.1)	2 (7.4)	27
		FF	1 (16.7)	2 (33.3)	3 (50)	6
	Total		23 (69.7)	5 (15.2)	5 (15.2)	33
9.00	RCSP	Straight	20 (71.4)	4 (14.3)	4 (14.3)	28
		FF	12 (70.6)	2 (11.8)	3 (17.6)	17
	Total		32 (71.1)	6 (13.3)	7 (15.6)	45
10.00	RCSP	Straight	0 (0)	0 (0)	0 (0)	0
		FF	2 (40)	0 (0)	3 (60)	5
	Total		2 (40)	0 (0)	3 (60)	5

*RCSP* rest calcaneal stance position, *FF* flat feet

A good intra-rater reliability for the RCSP was found; *ICC* (*ICC* = 0.81–0.87) and inter-rater reliability (*ICC* = 0.74).

## Discussion

The aim of this study was to determine the relationship between FPI and RCSP, in an effort to provide simple screening of paediatric FF, using an accessible, quick and low-cost tool for persons inexperienced in the intricacies of paediatric feet.

Paediatric FF continues to cause debate with little consensus regarding the assessment and consequence of this common clinical presentation [18]. Given this dissent and confusion, it is essential that clinical paediatric FF assessment be consistent, using validated measures. It is crucial to adopt an evidence-based approach, which can be problematic given the dearth of scientific certainty of many assessment methods [14].

A systematic review on the diagnosis of FF [14], analysed 20 measures of foot posture, including simple X-rays, footprint indices, static measures and plantar pressure. Of these, only Chippaux-Smirak index, Staheli arch index, and FPI had moderate validity, with high intra-observer and inter-observer reliability. Hence, the FPI is relevant for both researchers and doctors to assist better understanding of the paediatric FF.

More recent studies [16, 29] have utilised the FPI to ascertain normative data for paediatric foot posture, in children aged from 3 to 15 years. This large-scale dataset indicated that an FPI score of + 6 be considered a normal paediatric foot upper cut-point [18].

High correlation has been found for RCSP and heel valgus angles measured from radiographs [30] Together with the absence of radiation, RSCP can be realised as an accessible, safe and reliable tool to assess heel valgus as a FF constituent. Using RCSP instead of FPI provides a simpler FF screening mechanism, as recently proposed [20]. Previous research in children aged 8 to 13 years demonstrated moderate relationship between RCSP and FPI, with a kappa between 0.70 and 0.71 [20]. In contrast, our data returned a similar, if lower, relationship (*r* = 0.63; *p* < 0.01), in children aged 5 to 10 years.

This research distinguishes itself from previous studies by using the validated FPI to assess foot posture, rather than relying on the subjectivity of arch type, or indistinct soft tissue expansion as captured by the footprint indices of Staheli or Chippaux-Smirak. The FPI prioritises the three-dimensionality of the foot, as opposed to footprints.

The FPI, which is validated in children for foot type, was used as a ‘gold standard’ cut-off point for detection of paediatric FF, using the RCSP. The present study determined a RCSP score of 6 (degrees) as the optimal cut-off with moderate sensitivity (67%) and high specificity (85%). The cut-off value was analysed per gender, and the value of 6 was retained. Hence, the same cut-off value applies across both genders and both feet. Previous studies have used 4 (degrees) as a cut-off point for the RCSP in children [20, 31, 32], without evaluating the discriminant validity of this test. Our study, after calculating the ROC curve, discerned 6 (degrees) as the optimal cut-off point for the classification of FF in children. This has immediate application for future research, as well as for public health use.

The heterogeneity of detection of paediatric FF creates confusion for prevalence. A prevalence of 27% using footprints for the diagnosis of FF at age 6 to 10 years has been calculated [33]. Employing the FPI [20] found a prevalence of 26 to 29% at 8 to 13 years (using FPI + 6 for inclusion), and prevalence 23 to 24% from RCSP (cut-off point of 4) [20]. Our study found a lower FF prevalence using FPI (18.9%), and a higher prevalence with RCSP (29.6%). Notably, our study cut-off point for RCSP (6 degrees) was obtained from evaluating the specificity and sensitivity of the test and using the FPI as a ‘gold standard’.

In our study, we related BMI to FPI and to RCSP and found that body mass does not appear to exert significant influence on static foot posture. For many years, the relationship between obesity and FF represented a challenge due to the heterogeneity of FF classification tools [33, 34]. Several studies have reported that overweight and obesity affect the structure of the foot in children, such as the width of the midfoot [35] and presuming relationship FF. However, when comparing foot posture using the FPI, instead of a limited footprint, authors have found no clear relationship between BMI and foot posture [36].

The main limitation of this study is the age of participants, as only children between the ages of 5 and 10 were included, omitting those aged up to 14 years when bone development of the foot is complete. Another limitation was the ethnicity of participants (mostly Caucasian); hence, we cannot extrapolate these findings to population of other ethnicity. Furthermore, there was a small number of 10-year-old participants.

It is considered important to carry out investigations in the rest of the ages to compare the cut-off point. On the other hand, studies should also be carried out in other types of populations.

The clinical implication arising from this study are that the many physicians who encounter paediatric FF concerns can utilise the RCSP as a good screening tool, that is easy to use, quick, low cost and reliable. Knowing that the RCSP is a reasonable proxy for the FPI avails this simple tool for public health use, as part of basic paediatric lower limb triage, using the 3Q [37].

Undue attention to paediatric FF, which are usually flexible and painless, can cause unnecessary concern, result in unwarranted treatment and waste healthcare resources [14].

## Conclusion

Having obtained the RCSP cut-off point of 6 (degrees), with a sensitivity of 67%, and a specificity of 85%, using the FPI as ‘gold standard’, we have identified an important new use for an old tool, to facilitate screening for paediatric FF. The RCSP is especially useful for health professionals who are not specialised in paediatric feet or gait.

## References

[1] D’Amico JC (2009). Letter to the editor. J Am Podiatr Med Assoc.

[2] Evans AM, Nicholson H, Zakarias N (2009). The paediatric flat foot proforma (p-FFP): improved and abridged following a reproducibility study. J Foot Ankle Res.

[3] Labovitz JM (2006). The algorithmic approach to pediatric flexible pes planovalgus. Clin Podiatr Med Surg.

[4] Rome K, Ashford RL, Evans A (2010). Non-surgical interventions for paediatric pes planus. Cochrane Database Syst Rev.

[5] Michaudet C, Edenfield KM, Nicolette GW, Carek PJ (2018). Foot and ankle conditions: pes planus. FP Essent.

[6] Volpon JB (1994). Footprint analysis during the growth period. J Pediatr Orthop.

[7] Uden H, Scharfbillig R, Causby R (2017) The typically developing paediatric foot: how flat should it be? A systematic review. J Foot Ankle Res 10. 10.1186/S13047-017-0218-1 10.1186/s13047-017-0218-1 PMC5558233 28814975

[8] Riddiford-Harland DL, Steele JR, Baur LA (2011) Are the feet of obese children fat or flat? Revisiting the debate. Int J Obes 35(1):115–120. 10.1038/ijo.2010.119 10.1038/ijo.2010.119 20567243

[9] Pfeiffer M, Kotz R, Ledl T, Hauser G, Sluga M (2006). Prevalence of flat foot in preschool-aged children. Pediatrics.

[10] Harris EJ, Vanore JV, Thomas JL, Kravitz SR, Mendelson SA, Mendicino RW, Silvani SH, Gassen SC (2004). Diagnosis and treatment of pediatric flatfoot. J Foot Ankle Surg.

[11] Walczak M, Napiontek M (2003). Flexible flatfoot in children–a controversial subject. Chir Narzadow Ruchu Ortop Pol.

[12] Kothari A, Dixon PC, Stebbins J, Zavatsky AB, Theologis T (2015). The relationship between quality of life and foot function in children with flexible flatfeet. Gait Posture.

[13] Evans AM, Rome K, Carroll M, Hawke F (2022) Foot orthoses for treating paediatric flat feet. Cochrane Database Syst Rev. 10.1002/14651858.CD006311.PUB3 10.1002/14651858.CD006311.pub3 PMC8759438 35029841

[14] Banwell HA, Paris ME, Mackintosh S, Williams CM (2018). Paediatric flexible flat foot: how are we measuring it and are we getting it right? A systematic review. J Foot Ankle Res.

[15] Halabchi F, Mazaheri R, Mirshahi M, Abbasian L (2013). Pediatric flexible flatfoot; clinical aspects and algorithmic approach. Iran J Pediatr.

[16] Gijon-Nogueron G, Montes-Alguacil J, Alfageme-Garcia P, Cervera-Marin JA, Morales-Asencio JM, Martinez-Nova A (2016). Establishing normative foot posture index values for the paediatric population: a cross-sectional study. J Foot Ankle Res.

[17] Redmond AC, Crane YZ, Menz HB (2008) Normative values for the Foot Posture Index. J Foot Ankle Res 1. 10.1186/1757-1146-1-6 10.1186/1757-1146-1-6 PMC2553778 18822155

[18] Morrison SC, McClymont J, Price C, Nester C (2017) Time to revise our dialogue: how flat is the paediatric flatfoot? J Foot Ankle Res 10. 10.1186/S13047-017-0233-2 10.1186/s13047-017-0233-2 PMC5697058 29201146

[19] Gámez Guijarro M, Ortega Ávila AB, Gijón Noguerón G, Martínez Sebastián C (2021) Test de estudio biomecánico en niños y adolescentes: una revisión sistemática. Rev Española Podol 32. 10.20986/REVESPPOD.2021.1611/2021

[20] Cho Y, Park JW, Nam K (2019). The relationship between foot posture index and resting calcaneal stance position in elementary school students. Gait Posture.

[21] Sobradillo B, Aguirre A, Uresti U, Bilbao A, Fernández-Ramos C, Lizarraga A, Lorenzo H, Madariaga L, Rica I, Ruíz I et al (2004) Curvas y tablas de crecimiento. Estudios longitudinal y transversal. Bilbao: Fundación Faustino Orbegozo Eizaguirre; ISBN 8460799670

[22] Morrison SC, Ferrari J (2009). Inter-rater reliability of the Foot Posture Index (FPI-6) in the assessment of the paediatric foot. J Foot Ankle Res.

[23] Evans AM, Rome K, Peet L (2012). The foot posture index, ankle lunge test, Beighton scale and the lower limb assessment score in healthy children: a reliability study. J Foot Ankle Res.

[24] Root MI (1973). Biomechanical examination of the foot. J Am Podiatry Assoc.

[25] Sobel E, Levitz SJ, Caselli MA, Tran M, Lepore F, Lilja E, Sinaie M, Wain E (1999) Reevaluation of the relaxed calcaneal stance position. Reliability and normal values in children and adults. J Am Podiatr Med Assoc 89:258–264. 10.7547/87507315-89-5-258 10.7547/87507315-89-5-258 10349290

[26] Menz HB (2005). Analysis of paired data in physical therapy research: time to stop double-dipping?. J Orthop Sports Phys Ther.

[27] Menz HB (2004). Two feet, or one person? Problems associated with statistical analysis of paired data in foot and ankle medicine. Foot.

[28] Hanley JA, McNeil BJ (1982). The meaning and use of the area under a receiver operating characteristic (ROC) curve. Radiology.

[29] Gijon-Nogueron G, Martinez-Nova A, Alfageme-Garcia P, Montes-Alguacil J, Evans AM (2019) International normative data for paediatric foot posture assessment: a cross-sectional investigation. BMJ Open 9. 10.1136/BMJOPEN-2018-023341 10.1136/bmjopen-2018-023341 PMC6500282 30987983

[30] Lamm BM, Mendicino RW, Catanzariti AR, Hillstrom HJ (2005). Static rearfoot alignment: a comparison of clinical and radiographic measures. J Am Podiatr Med Assoc.

[31] Cho DJ, Ahn SY, Bok SK (2021). Effect of foot orthoses in children with symptomatic flexible flatfoot based on ultrasonography of the ankle invertor and evertor muscles. Ann Rehabil Med.

[32] Ahn SY, Bok SK, Kim BO, Park IS (2017). The effects of talus control foot orthoses in children with flexible flatfoot. J Am Podiatr Med Assoc.

[33] Pourghasem M, Kamali N, Farsi M, Soltanpour N (2016). Prevalence of flatfoot among school students and its relationship with BMI. Acta Orthop Traumatol Turc.

[34] Dowling AM, Steele JR, Baur LA (2001) Does obesity influence foot structure and plantar pressure patterns in prepubescent children? Int J Obes 25(6):845–852. 10.1038/sj.ijo.0801598 10.1038/sj.ijo.0801598 11439299

[35] Escalona-Marfil C, Prats-Puig A, Ortas-Deunosajut X, Font-Lladó R, Ruiz-Tarrazo X, Evans AM (2023). Children’s foot parameters and basic anthropometry — do arch height and midfoot width change?. Eur J Pediatr.

[36] Gijon-Nogueron G, Montes-Alguacil J, Martinez-Nova A, Alfageme-Garcia P, Cervera-Marin JA, Morales-Asencio JM (2017). Overweight, obesity and foot posture in children: a cross-sectional study. J Paediatr Child Health.

[37] Evans AM (2017). Mitigating clinician and community concerns about children’s flatfeet, intoeing gait, knock knees or bow legs. J Paediatr Child Health.

